# Surgical Deescalation Within Gynecologic Oncology

**DOI:** 10.1001/jamanetworkopen.2024.53604

**Published:** 2025-01-08

**Authors:** Alexa Kanbergs, Alexander Melamed, David Viveros-Carreño, Chi-Fang Wu, Roni Nitecki Wilke, Abigail Zamorano, Kimeera Paladugu, Laura Havrilesky, Jose Alejandro Rauh-Hain, Nuria Agusti

**Affiliations:** 1 Department of Gynecologic Oncology and Reproductive Medicine, The University of Texas MD Anderson Cancer Center, Houston; 2 Department of Obstetrics and Gynecology, Massachusetts General Hospital, Boston; 3 Unidad Ginecología Oncológica, Grupo de Investigación GIGA, Centro de Tratamiento e Investigación Sobre Cáncer Luis Carlos Sarmiento Angulo, Bogotá, Colombia; 4 Clínica Universitaria Colombia, Bogotá, Colombia; 5 Department of Health Services Research, The University of Texas MD Anderson Cancer Center, Houston; 6 Division of Gynecologic Oncology, Department of Obstetrics, Gynecology, and Reproductive Health Sciences, The University of Texas Health Science Center at Houston, Houston; 7 University of Houston, Houston, Texas; 8 Department of Obstetrics and Gynecology, Duke University Medical Center, Durham, North Carolina

## Abstract

**Importance:**

The goal of surgical deescalation is to minimize tissue damage, enhance patient outcomes, and reduce the adverse effects often associated with extensive or traditional surgical procedures. This shift toward less invasive techniques has the potential to revolutionize surgical practices, profoundly impacting the methods and training of future surgeons.

**Objective:**

To evaluate adoption of surgical deescalation within the field of gynecologic oncology using The National Cancer Database.

**Design, Setting, and Participants:**

This cohort study used prospectively collected data from the National Cancer Database from January 2004 to December 2020. Eligible participants included women in the US who received a diagnosis of clinical stage I to IV endometrial, ovarian, cervical, or vulvar cancer within this time frame. Data were analyzed between January and June 2024.

**Exposure:**

Diagnosis of stage I to IV endometrial, ovarian, cervical, or vulvar cancer.

**Main Outcomes and Measures:**

The primary outcome was surgical deescalation, which included evaluation of receipt of surgical intervention, the surgical approach, the type of lymph node assessment, and salvage interventions for disease-affected organs. A Poisson model was applied to estimate the average annual percentage change (AAPC) in the receipt of surgical treatment.

**Results:**

A total of 1 218 490 patients (mean [SD] age at diagnosis, 61.2 [13.7] years) were included. Over the study period, the percentage of patients undergoing surgery decreased from 47.4% to 39.9% for those with cervical cancer (AAPC, −1.3%; 95% CI, −1.6% to −1.1%), from 72.0% to 67.9% for those with ovarian cancer (AAPC, −0.5%; 95% CI, −0.6% to −0.4%), from 83.7% to 79.1% for those with endometrial cancer (AAPC, −0.5%; 95% CI, −0.7% to 11 −0.4%), and from 81.1% to 72.6% for those with vulvar cancer (AAPC, −1.3%; 95% CI, −1.6% to −0.9%). The use of minimally invasive surgery increased from 45.8% to 82.2% for those with endometrial cancer (AAPC, 4.6%; 95% CI, 4.5% to 4.8%) and from 13.3% to 37.0% for those with ovarian cancer (AAPC, 9.4%; 95% CI, 9.0% to 9.7%). Sentinel lymph node dissection increased from 0.7% to 39.6% for patients with endometrial cancer (AAPC, 51.8%; 95% CI, 50.5% to 53.2%), from 0.2% to 10.6% for patients with cervical cancer (AAPC, 44.0%; 95% CI, 39.3% to 48.9%), and from 12.3% to 36.9% for patients with vulvar cancer (AAPC, 10.7%; 95% CI, 8.0% to 13.5%) cancers, whereas the rate of complete lymphadenectomies decreased in all 3 groups. The rate of fertility-sparing surgery for patients with cervical cancer younger than 40 years rose from 17.8% to 28.1% (AAPC, 3.1%; 95% CI, 2.3%-3.9%).

**Conclusions and Relevance:**

These findings suggest that over the past 15 years, the field of gynecologic oncology has moved toward surgical deescalation through an overall reduction in the number of patients who undergo surgery, increased use of minimally invasive surgical techniques, and increased use of sentinel lymph node techniques. Future research should focus not only on understanding the impact of surgical escalation on patients (including disease outcomes, quality of life, and equitable access to these services), but also on surgical training.

## Key Points

**Question:**

How has surgical deescalation evolved in gynecologic oncology?

**Findings:**

In this cohort study of 1 218 490 patients with cervical, endometrial, ovarian, or vulvar cancer, several areas of surgical deescalation were identified, including an overall decrease in surgical operations, the adoption of minimally invasive techniques, and a shift toward sentinel lymph node evaluation over complete lymphadenectomy.

**Meaning:**

These findings suggest that as a field, gynecologic oncology is moving towards less invasive surgical methods, which may lead to potential benefits for patients but also raises concerns for surgical training and the equitable distribution of these services.

## Teaser

This cohort study examines how surgical management practices have evolved over time within the field of gynecologic oncology.

## Introduction

Deescalation, as per the European Society for Medical Oncology Precision Medicine Working Group,^1^ refers to omitting a segment of standard treatment, shortening the duration of treatment, or both while maintaining survival rates and reducing patient burden. The concept of deescalation has already gained substantial ground in medical oncology,^2,3,4,5,6,7^ and surgical deescalation has also emerged as a strategy across surgical subspecialties to optimize patient outcomes while reducing the adverse effects associated with extensive surgical interventions and improving patient quality of life.^8,9,10^

For the treatment of gynecologic cancers, surgical deescalation refers to procedures aimed at minimizing tissue injury while preserving tissue integrity during cancer-related operations. Over the past decade, studies have validated deescalation approaches for various gynecological cancers and procedures, such as the adoption of laparoscopic technique,^11,12,13^ sentinel lymph node (SLN) biopsies,^14,15,16,17^ and limiting organ removal or resection to only those necessary to maintain oncologic outcomes.^18,19^ However, none, to our knowledge, have performed a comprehensive analysis of surgical deescalation trends across the surgical spectrum offered by gynecologic oncologists.

A broader analysis of these trends could help in understanding the general direction of deescalation practices and provide a basis for developing hypotheses regarding their effectiveness and potential benefits. In this study, we used a national cancer registry to identify how surgical management practices have evolved over time within the field of gynecologic oncology.

## Methods

This cohort study was approved by the MD Anderson institutional review board with a waiver of informed consent because it was not feasible to obtain consent from the large number of individuals within the national cancer database. The reporting of this study followed the Strengthening the Reporting of Observational Studies in Epidemiology (STROBE) reporting guideline. We used data prospectively collected from the National Cancer Database (NCDB), a joint project by the American College of Surgeons and the American Cancer Society that collects data on approximately 70% of patients with incident cancer in the US.^20^ The database contains data from more than 1500 Commission on Cancer–accredited facilities. Using this database, we identified all women who received a diagnosis of clinical stage (I-IV) endometrial, ovarian, cervical, or vulvar cancer from January 1, 2004, to December 31, 2020. Diseases were categorized according to the *International Classification of Diseases for Oncology*, *Third Edition*, and staged according to the American Joint Committee on Cancer Staging Manual, seventh and eighth editions.^21,22^ The analysis included patients with cancers at all stages and histologic grades unless otherwise specified. Patients with synchronous or prior malignant neoplasms or those with noninvasive disease were excluded. Cohorts for analysis were generally identified based on the National Comprehensive Cancer Network Clinical Practice Guidelines in Oncology or expert recommendations. eTable 1 in Supplement 1 provides a detailed overview of and rationale for each cohort and eTable 2 in Supplement 1 provides the specific coding criteria applied to each cohort for analysis purposes. Patients with missing variables used to define each cohort were excluded from analysis. The beginning of the year-range used for each analysis was based on the earliest year that data were available for the specified coding criteria.

### Defining Surgical Deescalation

For our study, we defined surgical deescalation as the use of less invasive surgical approaches or reduction of the extent of surgery (eg, removing fewer organs, anatomical structures, and/or lymph nodes). Because the definition of surgical deescalation is not standardized, we chose this definition to encompass a diverse range of strategies aimed at preserving precancer anatomy and minimizing surgical radicality while maintaining effective treatment outcomes. Based on this definition, we created several cohorts to evaluate trends in minimally invasive surgery (MIS; ie, laparoscopy and robotic surgery), SLN biopsy (as an alternative to complete lymphadenectomy), and limiting organ removal or resection to only those necessary to maintain oncologic outcomes.

#### MIS

To identify deescalation trends in MIS, we evaluated patients with cervical, endometrial, or ovarian cancer who underwent surgical treatment of disease at any stage or of any histologic grade. Given variations in clinical practice based on disease stage, we also performed supplementary evaluations of trends in surgical approaches based on early or late disease stages.

#### SLN Evaluation

Patients with cervical, endometrial, or vulvar cancer were placed into cohorts: (1) those who underwent SLN evaluation exclusively and (2) those who underwent a lymphadenectomy regardless of a preceding SLN dissection (SLND). We performed the initial analyses using the NCDB variable, scope of regional lymph, which identifies the removal, biopsy analysis, or aspiration of regional lymph nodes at the time of surgery of the primary site or during a separate surgical event. Given potential underreporting of this variable, we validated these results by performing additional analyses using the NCDB variable, regional lymph nodes examined, which documents the total number of regional lymph nodes removed and examined by pathologists. To achieve the highest specificity results and avoid misidentifying an SLND as a lymphadenectomy, we defined SLND as the examination of fewer than 4 lymph nodes and lymphadenectomy as the examination of 4 or more lymph nodes. The specificity rate of this approach ranged from 90% to 97% depending on the cancer site. Notably, the approach to lymph node dissection was not evaluated for patients with ovarian cancer because SLND is not considered standard of care for these patients. We also assessed trends in para-aortic lymph node dissection among patients with cervical cancer of any stage and patients with endometrial cancer with clinical stage I and II disease stratified into 3 groups: (1) stage IA endometrioid histologic grade 1 or 2, (2) stage IB endometrioid histologic grade 1 or 2, and (3) stage I to II with high-risk histologic grade.

#### Surgical Radicality

The evaluation of surgical radicality encompassed the identification of organs and/or structures preserved during the surgical management of individual cancer types, alongside an assessment of trends in fertility-sparing surgery (FSS). Owing to the varied nature of surgical techniques and the extent of surgery within each cancer subtype, a standardized definition for preserved organs and structures was not feasible. Instead, cohorts for analysis were determined based on clinical guidelines or expert recommendations (eTable 1 in Supplement 1).

Among patients with cervical cancer, we evaluated trends in the use of radical vs simple hysterectomy for patients with low-risk early-stage disease. Because of the limits of the NCDB coding, we were unable to evaluate trends in the extent of surgery for patients with ovarian cancer. Specifically, codes for splenectomy, hepatectomy, and pancreatectomy were not available for gynecologic procedures, and codes for bowel surgery and urologic surgery did not adequately capture the differences in extent of surgery.

Among patients with endometrial cancer, we evaluated ovarian preservation for patients with clinical stage I disease who had endometrial histologic findings, who were younger than 40 years, and who underwent hysterectomy. We set this age criterion to capture trends for patients most likely to benefit from ovarian preservation. FSS, defined as retention of the uterus and at least 1 ovary, was included in our analysis of trends in ovarian and cervical cancer. For patients with cervical cancer, we included those younger than 40 years with stage IA1 to IB1 disease and select patients with stage IB2 disease and tumor sizes smaller than 2 cm who underwent FSS or a hysterectomy. In addition, considering variations in pregnancy rates, we conducted subanalyses across different age groups to evaluate trends. For patients with ovarian cancer, those with clinical stage IA or IC disease who were younger than 40 years were included.

### Statistical Analysis

Time trends in the percentage of patients receiving interventions were assessed using the Cochran-Armitage trend test. A Poisson model was applied to estimate the average annual percentage change (AAPC) in the receipt of surgical treatment. To account for temporal trends, the model was adjusted for the year of diagnosis, which was treated as a continuous variable. SAS Enterprise Guide version 7.1 (SAS Institute) was used for all statistical analyses. The threshold for statistical significance was a 2-sided *P* < .05. Data analysis took place between January and June 2024.

## Results

We identified 1 218 490 patients (mean [SD] age at diagnosis, 61.2 [13.7] years; 130 137 Black [10.7%]; 87 816 Hispanic [7.2%]; 934 735 non-Hispanic White [76.7%]), including 166 779 with cervical cancer (13.7%), 686 458 with endometrial cancer (56.3%), 301 123 with ovarian cancer (24.7%), and 64 130 with vulvar cancer (5.2%) from January 1, 2004, to December 31, 2020, in the NCDB database. Most participants were insured (552 499 with private insurance [45.3%] and 595 245 [48.8%] with public insurance), had stage I disease (624 922 participants [51.3%]), lived in metropolitan areas (995 252 participants [81.7%]), and received care at either an academic center (488 190 participants [40.1%]) or a community program (427 862 participants [35.1%]). Detailed patient characteristics for the entire study population and by disease site are provided in eTable 3 in Supplement 1.

**Figure 1.**
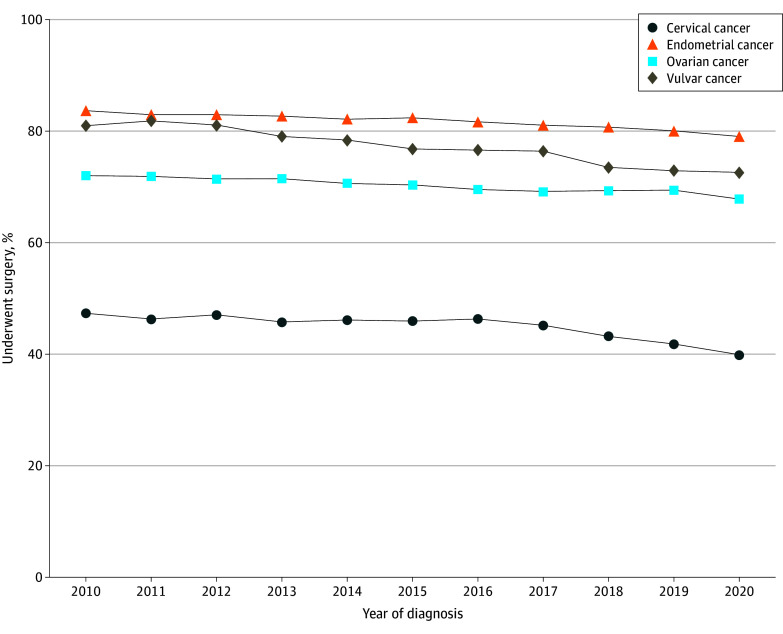
Patients Who Underwent Surgical Treatment of Gynecologic Cancer

Overall, the percentage of patients undergoing any surgical treatment (MIS, open, or converted) decreased from 2010 to 2020 (ie, the end of the study period). Namely, the percentage of patients who underwent surgery decreased from 47.4% to 39.9% for those with cervical cancer (AAPC, −1.3%; 95% CI, −1.6% to −1.1%), from 72.0% to 67.9% for those with ovarian cancer (AAPC, −0.5%; 95% CI, −0.6% to −0.4%), from 83.7% to 79.1% for those with endometrial cancer (AAPC, −0.5%; 95% CI, −0.7% to −0.4%), and from 81.1% to 72.6% for those with vulvar cancer (AAPC, −1.3%; 95% CI, −1.6% to −0.9%) (Figure 1). Time trends stratified by early and late-stage disease are included in eFigure 1 in Supplement 1.

### MIS

**Figure 2.**
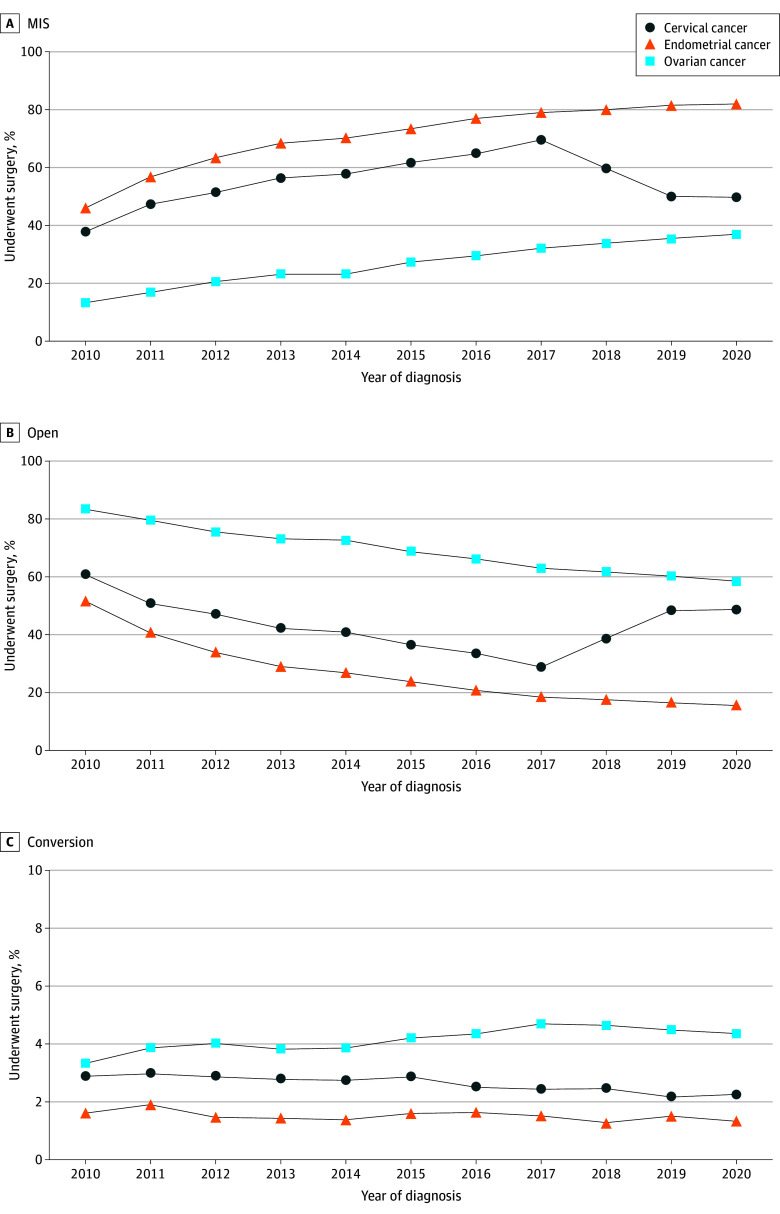
Surgical Approaches Used Among Patients With Cervical, Endometrial, or Ovarian Cancer MIS indicates minimally invasive surgery.

From 2010 to 2020, the use of MIS for patients who underwent any surgical treatment, regardless of cancer stage, increased from 45.8% to 82.2% for those with endometrial cancer (AAPC, 4.6%; 95% CI, 4.5%-4.8%) and from 13.3% to 37.0% for those with ovarian cancer (AAPC, 9.4%; 95% CI, 9.0%-9.7%) (Figure 2). Conversely, the use of laparoscopy for cervical cancer treatment fluctuated, initially rising to a peak of 69.7% in 2017, before declining to 49.9% in 2020 (AAPC, 2.4%; 95% CI, 2.0%-2.8%) (Figure 2). Further analyses of MIS use stratified by early-stage (stage I and II) and late-stage (stage III and IV) disease demonstrated similar and consistent trends as detailed in eFigure 2 and eFigure 3 in Supplement 1.

### Lymph Node Evaluation

#### Cervical Cancer

**Figure 3.**
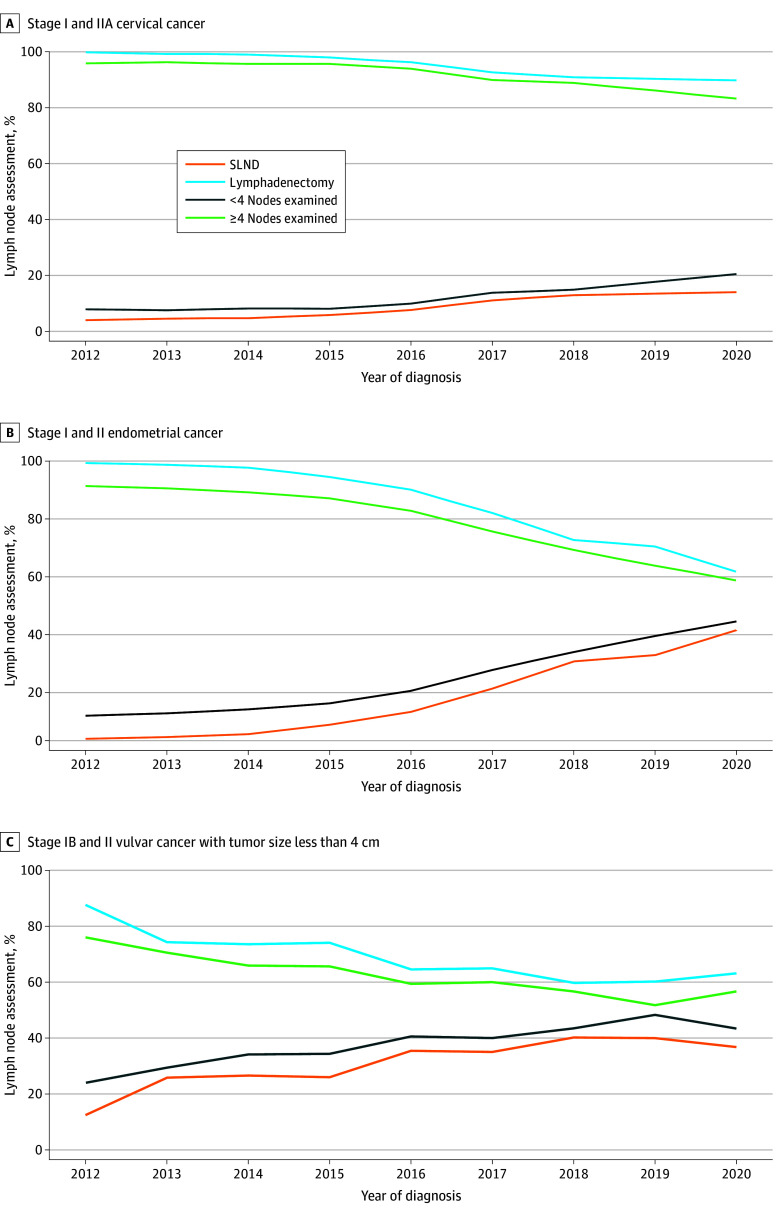
Methods of Lymph Node Assessment Used Among Patients SLND indicates sentinel lymph node dissection.

For patients with clinical stage I or IIA cervical cancer who underwent lymph node dissection from 2012 to 2020, the rate of SLND increased from 0.2% to 10.6% (AAPC, 44.0%; 95% CI, 39.3% to 48.9%), and the rate of complete lymphadenectomy decreased from 99.7% to 89.3% (AAPC, −1.6%; 95% CI, −2.2% to −1.0%). When using the number of lymph nodes dissected (<4 vs ≥4) as a proxy for SLND and lymphadenectomy, the percentage of patients with fewer than 4 lymph nodes evaluated increased from 2004 to 2020, rising from 4.2% to 17.4% (AAPC, 23.9%; 95% CI, 21.2% to 26.6%), whereas the percentage of patients with 4 or more lymph nodes evaluated decreased from 95.8% to 82.6% from 2004 to 2020 (AAPC, −1.6%; 95% CI, −2.2% to −1.0%). (Figure 3A). Additionally, the frequency of patients (with any stage cancer) who underwent pelvic and para-aortic lymph node dissection decreased from 2012 to 2017, going from 11.9% to 6.1% (AAPC, −12.6%; 95% CI, −13.9% to −11.1%) (eFigure 4 in Supplement 1).

#### Endometrial Cancer

Among patients with clinical stage I or II endometrial cancer who underwent lymph node assessment, the rate of SLND increased from 2012 to 2020, rising from 0.7% to 39.6% (AAPC, 51.8%; 95% CI, 50.5% to 53.2%), whereas the rate of complete lymphadenectomy decreased from 99.3% to 60.4% (AAPC, −5.8%; 95% CI, −6.0% to −5.5%) in the same period. Similarly, when analyzing the number of lymph nodes removed within the same cohort, the percentage of patients who had fewer than 4 lymph nodes removed from 2004 to 2020 increased from 8.9% to 42.7% (AAPC, 14.1%; 95% CI, 13.8% to 14.5%) (Figure 3B). Around 2016, we observed the most rapid increase in the use of SLND for endometrial cancer. Additionally, we evaluated the removal of pelvic lymph nodes vs pelvic and para-aortic lymph nodes for patients with clinical stage I or II endometrial cancer stratified by risk of metastasis (with group 1 comprising patients with endometrioid histologic grade 1 or 2 and stage IA cancer, group 2 comprising patients with endometrioid histologic grade 1 or 2 and stage IB cancer, and group 3 comprising patients with endometrioid histologic grade 3 or other high risk histologic grade and stage I or II cancer) (eFigure 5 in Supplement 1). From 2012 to 2017, the number of patients undergoing pelvic and para-aortic lymph node dissections decreased for group 1 (17.4% to 10.3%; AAPC, −10.6%; 95% CI, −12.5% to −8.7%), group 2 (31.0% to 20.1; AAPC, −8.1.%; 95% CI, −11.0% to −5.0%), and group 3 (49.0% to 38.3%; AAPC, −2.2%; 95% CI, −7.2% to −3.0%).

#### Vulvar Cancer

Among patients with clinical stage IB or stage II vulvar cancer with a tumor size smaller than 4 cm who underwent lymph node assessment, the rate of SLND increased from 2012 to 2020, going from 12.3% to 36.9% (AAPC, 10.7%; 95% CI, 8.0% to 13.5%). In the same time frame, the rate of complete lymphadenectomy decreased from 87.7% to 63.1% (AAPC, −4.3%; 95% CI −5.9% to −2.8%). Similarly, when analyzing the number of lymph nodes removed within the same cohort, the percentage of patients who had fewer than 4 lymph nodes removed increased from 2012 to 2020, rising from 24.0% to 43.4% (AAPC, 10.2%; 95% CI, 8.9% to 11.5%) (Figure 3C).

### Radicality of Surgery

#### Cervical Cancer

Among patients with invasive histologic findings who underwent lymph node assessment and any type of hysterectomy for clinical stage IA2 or IB1 disease with a tumor size smaller than 2 cm, the rate of extended procedures (ie, radical hysterectomy) increased from 2012 to 2020, going from 58.1% to 68.8% (AAPC, 2.0%; 95% CI, 0.0% to 4.0%), whereas the rate of simple hysterectomy declined from 42.0% to 31.2% (AAPC, −2.8%; 95% CI, −5.2% to −0.4%) (eFigure 6 and eTable 4 in Supplement 1). Besides the change in the type of hysterectomy, we also noticed a decrease in the total number of hysterectomies performed for cervical cancer during this period. Specifically, radical hysterectomies decreased from 173 cases in 2012 to 141 cases in 2020.

**Figure 4.**
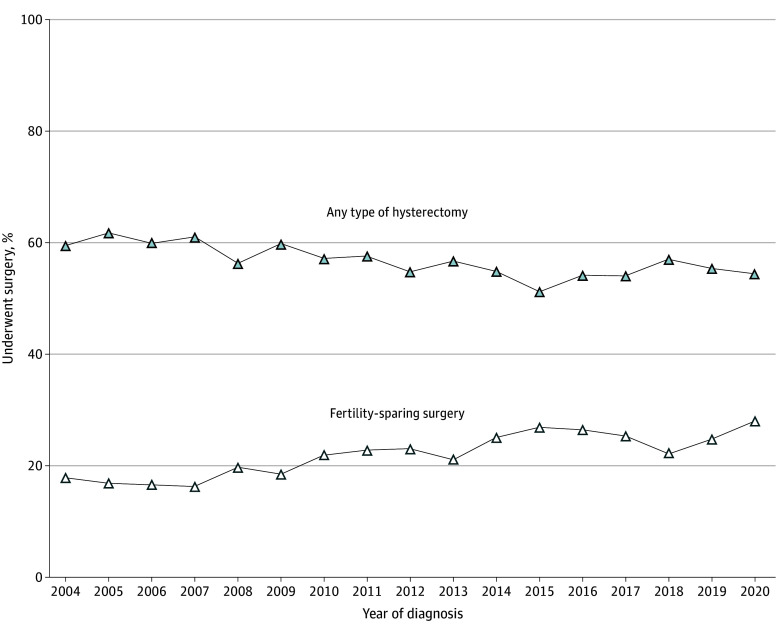
Fertility-Sparing Surgery vs Hysterectomy This comparison includes patients with cervical cancer and a tumor size smaller than 2 cm who were younger than 40 years.

The number of patients with cervical cancer who underwent FSS, were younger than 40 years, had a tumor size smaller than 2 cm, and underwent surgical treatment (hysterectomy vs conization or trachelectomy) increased from 2004 to 2020, going from 17.8% to 28.1% (AAPC, 3.1%; 95% CI, 2.3%-3.9%) (Figure 4). This trend was even more pronounced among patients aged 25 to 35 years, in whom the FSS rate from 2004 to 2020 increased from 21.9% to 34.4% (AAPC, 3.2%; 95% CI, 2.2%-4.1%) (eFigure 7 in Supplement 1).

#### Endometrial Cancer and Ovarian Cancer

**Figure 5.**
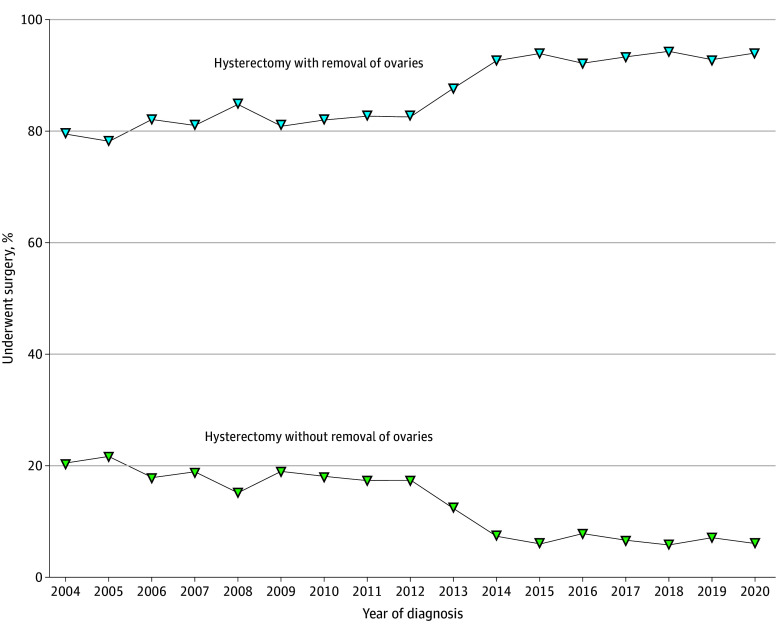
Rates of Hysterectomy With vs Without Removal of Ovaries This comparison includes patients younger than 40 years with clinical stage IA endometrial cancer and endometrioid histological examination (excluding those with grade 3 disease).

Among patients with stage I endometrial cancer and endometrioid histologic grade 1 or 2 who were younger than 40 years, the rate of ovarian preservation during hysterectomy declined from 2004 to 2020, going from 20.6% to 6.0% (AAPC, −8.8%; 95% CI, −11.1% to −6.3%). In contrast, the rate of ovarian removal during hysterectomy increased from 2004 to 2020, rising from 79.5% to 94.0% (AAPC, 1.3%; 95% CI, 0.3% to 2.3%) (Figure 5). Among patients younger than 40 years with stage IA or IC ovarian cancer who underwent surgery, the percentage who received FSS vacillated greatly over time and did not demonstrate a clear trend (eFigure 8 in Supplement 1).

## Discussion

In this cohort study using a national database, we examined trends in surgical deescalation in gynecologic oncology. We identified several deescalation trends, including a decrease in surgical operations, a significant adoption of MIS, and a shift toward SLN evaluation instead of complete lymphadenectomy. However, we observed varying levels of deescalation in surgical radicality. While these trends are promising for patient quality of life, they also have profound implications for surgical trainees and junior attendings working in this evolving landscape.

Our study observed a decline in the number of patients undergoing surgery for cancer management across all cancer subtypes. We suspect that these findings may reflect (1) an increased use of neoadjuvant chemotherapy, which could lead to identifying patients who do not respond to chemotherapy and as a result either do not proceed to surgery or die before surgery can be performed or (2) increased use of alternative up-front treatment modalities such as chemoradiation. However, further research is needed to identify the factors underlying this trend.

For patients who underwent surgical treatment, ovarian and endometrial cancers were increasingly managed with MIS for patients with early- and late-stage disease. The increased use of MIS for patients with endometrial cancer reflects the findings of numerous randomized trials that support equivalent oncologic outcomes with a laparoscopic approach.^12,23^ The management of ovarian cancer has also increasingly moved toward a laparoscopic approach, even for advanced-stage disease. However, prospective randomized trials, such as Nitecki et al,^24^ are ongoing to assess noninferiority of MIS for advanced-stage ovarian cancer after neoadjuvant chemotherapy. Conversely, for cervical cancer, MIS use increased steadily from 2010 to 2017, which was followed by an abrupt decline after publication of the Ramirez et al trial,^25^ which demonstrated shorter overall survival with minimally invasive radical hysterectomy than with open surgery for patients with stage IA2 to IB1 disease.

We observed a significant increase in SLN evaluation, along with a corresponding decrease in complete lymphadenectomy among patients with endometrial, vulvar, and cervical cancers. This trend mirrors similar shifts in the management of other cancers, such as breast and melanoma, indicating a broader move toward more tailored and less invasive lymph node evaluation procedures.^26,27,28^ In gynecologic oncology, this less invasive approach to nodal evaluation is supported by evidence from randomized clinical trials such as Rossi et al^14^ for endometrial cancer and Levenbeck et al^15^ for vulvar cancer. However, the uptake of SLND evaluation for cervical cancer has not been as pronounced, likely due to the limited number of studies on oncologic outcomes published before 2020.

Regarding para-aortic lymph node dissection, we observed a decrease in the percentage of patients undergoing para-aortic lymphadenectomy for early-stage endometrial cancer, particularly those with low-risk disease. For ovarian cancer, the rates of pelvic and para-aortic lymphadenectomy have remained relatively stable. However, the 2019 trial by Harter et al,^29^ which found no benefit in overall or progression-free survival from systematic pelvic and para-aortic lymphadenectomy in advanced ovarian cancer, may result in a decline in these procedures.

Unlike the consistent rise in MIS and SLN evaluation, less radical surgical approaches had varied adoption. For endometrial cancer, ovarian preservation during hysterectomy declined in patients younger than 40 years with stage IA low-risk disease, mirroring findings from Wright et al^30^ from 1998 to 2022, which showed most young women still undergo oophorectomy. For cervical cancer, we found increased use of FSS, possibly owing to emerging evidence of its safety and equivalent oncologic outcomes to standard surgery for select patients with early-stage cervical cancer^31^ and a national trend of delayed childbearing.^32^ We also observed a higher percentage of patients with IA2 and IB1 cervical cancer undergoing radical hysterectomy compared with simple hysterectomy. However, the overall number of radical hysterectomies performed decreased, which aligns with the findings of Matsuo et al^33^ from the Surveillance, Epidemiology, and End Results database. We anticipate this trend will only be amplified with the recent publication of findings from trials by Schmeler et al^34^ and Plante et al.^35^

The trends toward surgical deescalation in gynecologic oncology are mirrored in other surgical specialties. Bingmer et al^36^ found a 34.9% decrease in the mean number of open procedures performed by general surgery residents from 1999 to 2018. Similarly, Smith et al^37^ noted a 38.0% decrease in the total open aortic repair volume among vascular surgery trainees, with a corresponding decrease in the median number of such procedures performed by senior trainees. Although surgical deescalation has many potential benefits for patients, it raises concerns about the ability and comfort of graduating trainees and junior faculty to safely perform less common procedures. In a survey by Nguyen et al^38^ that included gynecologic oncologists who graduated from 2015 to 2020, only 76% felt comfortable performing a radical hysterectomy and only 60% felt comfortable performing a laparoscopic para-aortic lymphadenectomy. Respondents also reported greater comfort performing an inguinal SLND when compared with an inguinal femoral lymph node dissection (82% vs 70%, respectively).^38^ Many trainees felt uncomfortable performing ultraradical procedures, such as pelvic exenterations, low anterior resections, splenectomy, and ureteral procedures.^38^ Surveys of other surgical trainees in different specialties indicated similar trends. In a national survey of vascular surgery trainees and junior attendings,^39^ authors reported a paucity of complex open vascular cases corresponding to reduced comfort in performing these procedures. Providing innovative educational opportunities to simulate these procedures so that trainees can gain necessary skills is imperative.

Beyond focusing on the impact of deescalation on surgical trainees, future research should explore the corresponding increased use of alternative therapeutic modalities in place of surgery, such as chemotherapy, radiation therapy, and advanced targeted therapies, including immunotherapy and molecularly targeted treatments. Additionally, future studies should examine the impact of deescalation on disparities in cancer care. As novel surgical techniques emerge and standards of care evolve, a crucial step will be to analyze the distribution of these services and correlate them with patient outcomes. Prior research demonstrated that MIS for hysterectomy in patients with early-stage endometrial cancer is more common among White, privately insured patients receiving care at high-volume surgical centers.^40^ Similar disparities have been documented in the use of SLN evaluation.^41^

### Limitations

Although our study benefited from a large cohort of patients, we acknowledge the limitations inherent to use of retrospective databases, namely, potential inaccuracies, variability, and underreporting in surgical and treatment codes and confounding factors that are not accounted for. In instances of underreporting, we employed alternative analyses to validate our findings. Additionally, we did not perform some analyses that would have contributed to understanding of surgical trends owing to the limited codes available for specific disease sites. For example, we were unable to evaluate trends in the radicality of cytoreductive surgery in ovarian cancer (ie, changes in frequency of bowel resections, urinary diversions, or splenectomies) because those variables are not available for gynecologic procedures within the NCDB data dictionary.

## Conclusions

This cohort study illustrates a trend toward deescalation in gynecologic oncology, characterized by the adoption MIS and SLN evaluation. Emerging evidence suggesting oncologic adequacy and lower morbidity may be underlying these trends. However, variations persist in the extent and consistency of their application, particularly concerning the radicality of surgical procedures, which may evolve as new trial data become available. This shift raises important considerations and invites further exploration into its implications for cancer treatment, surgeon training, and the equitable accessibility of care. Continued research appears essential to understand its impact on patient outcomes, training standards, and equitable access to these advanced surgical methods.

## Notes

Supplement 1. **eTable 1.** Cohorts Included in Our Analyses and the Rationale for Their Selection **eTable 2.** NCBD Variables/Codes Used in Study Analyses **eTable 3.** Patient Characteristics **eFigure 1.** Patients Who Underwent Surgical Treatment of Gynecologic Cancer Stratified by Early (Stage I and II) and Late (Stage III and IV) Stage **eFigure 2.** Minimally Invasive Surgery (MIS), Open Surgery, or Conversion-to-Open Surgery (Conversion) Among Patients With Cervical, Endometrial, or Ovarian Cancer and Stage I or II Disease **eFigure 3.** Minimally Invasive Surgery (MIS), Open Surgery, or Conversion-to-Open Surgery (Conversion) Among Patients With Cervical, Endometrial, or Ovarian Cancer and Stage III or IV Disease **eFigure 4.** Pelvic or Pelvic + Para-Aortic Lymph Node Dissection in Patients With Any Stage Cervical Cancer **eFigure 5.** Para-Aortic Lymph Node Assessment in Patients With Early-Stage Endometrial Cancer Stratified by Risk Factors for Nodal Metastasis **eFigure 6.** Simple vs Radical (Extended) Hysterectomy in Patients With IA2 and IB1 Cervical Cancer With a Tumor Smaller Than 2 cm **eTable 4.** The Raw Data for Graph 7A **eFigure 7.** Fertility-Sparing Surgery Versus Hysterectomy in Patients With Cervical Cancer and a Tumor Size Smaller Than 2 cm and Who Were 25 to 35 Years Old **eFigure 8.** Fertility-Sparing Versus Non-Fertility-Sparing Treatment in Patients With Clinical Stage IA or IC Ovarian Cancer Aged Less 40

Supplement 2.
Data Sharing Statement
