# Error in Byline

**DOI:** 10.1001/jamanetworkopen.2025.0959

**Published:** 2025-02-10

In the Original Investigation titled “Surgical Deescalation Within Gynecologic Oncology,”^1^ published January 8, 2025, there was an error in the byline, in which coauthor Kimeera Paladugu’s name was mispelled. This article has been corrected.^1^
