# Error in Figure

**DOI:** 10.1001/jamanetworkopen.2025.4391

**Published:** 2025-03-10

In the Original Investigation titled “Surgical Deescalation Within Gynecologic Oncology,”^1^ published January 8, 2025, there was an error in Figure 2, in which the plots for cervical and endometrial cancer were mislabeled. This article has been corrected.^1^

## References

[1] Kanbergs A, Melamed A, Viveros-Carreño D, . Surgical deescalation within gynecologic oncology. JAMA Netw Open. 2025;8(1):e2453604. doi:10.1001/jamanetworkopen.2024.53604 39775807 PMC11811805

