# Supplementary material for: Surgical Deescalation Within Gynecologic Oncology
Source: JAMA Netw Open. 2025 Jan 8;8(1):e2453604. doi: 10.1001/jamanetworkopen.2024.53604 (PMC11811805; doi:10.1001/jamanetworkopen.2024.53604)
Supplement: Supplement 1. — eTable 1. Cohorts Included in Our Analyses and the Rationale for Their Selection eTable 2. NCBD Variables/Codes Used in Study Analyses eTable 3. Patient Characteristics eFigure 1. Patients Who Underwent Surgical Treatment of Gynecologic Cancer Stratified by Early (Stage I and II) and Late (Stage III and IV) Stage eFigure 2. Minimally Invasive Surgery (MIS), Open Surgery, or Conversion-to-Open Surgery (Conversion) Among Patients With Cervical, Endometrial, or Ovarian Cancer and Stage I or II Disease eFigure 3. Minimally Invasive Surgery (MIS), Open Surgery, or Conversion-to-Open Surgery (Conversion) Among Patients With Cervical, Endometrial, or Ovarian Cancer and Stage III or IV Disease eFigure 4. Pelvic or Pelvic + Para-Aortic Lymph Node Dissection in Patients With Any Stage Cervical Cancer eFigure 5. Para-Aortic Lymph Node Assessment in Patients With Early-Stage Endometrial Cancer Stratified by Risk Factors for Nodal Metastasis eFigure 6. Simple vs Radical (Extended) Hysterectomy in Patients With IA2 and IB1 Cervical Cancer With a Tumor Smaller Than 2 cm eTable 4. The Raw Data for Graph 7A eFigure 7. Fertility-Sparing Surgery Versus Hysterectomy in Patients With Cervical Cancer and a Tumor Size Smaller Than 2 cm and Who Were 25 to 35 Years Old eFigure 8. Fertility-Sparing Versus Non-Fertility-Sparing Treatment in Patients With Clinical Stage IA or IC Ovarian Cancer Aged Less 40 [file jamanetwopen-e2453604-s001.pdf]

Supplemental Online Content

Kanbergs A, Melamed A, Viveros-Carreño, et al. Surgical deescalation within gynecologic oncology. *JAMA Netw. Open*. 2025;8(1):e2453604. doi:10.1001/jamanetworkopen.2024.53604

eTable 1. Cohorts Included in Our Analyses and the Rationale for Their Selection
eTable 2. NCBD Variables/Codes Used in Study Analyses
eTable 3. Patient Characteristics
eFigure 1. Patients Who Underwent Surgical Treatment of Gynecologic Cancer Stratified by Early (Stage I and II) and Late (Stage III and IV) Stage
eFigure 2. Minimally Invasive Surgery (MIS), Open Surgery, or Conversion-to-Open Surgery (Conversion) Among Patients With Cervical, Endometrial, or Ovarian Cancer and Stage I or II Disease
eFigure 3. Minimally Invasive Surgery (MIS), Open Surgery, or Conversion-to-Open Surgery (Conversion) Among Patients With Cervical, Endometrial, or Ovarian Cancer and Stage III or IV Disease
eFigure 4. Pelvic or Pelvic + Para-Aortic Lymph Node Dissection in Patients With Any Stage Cervical Cancer
eFigure 5. Para-Aortic Lymph Node Assessment in Patients With Early-Stage Endometrial Cancer Stratified by Risk Factors for Nodal Metastasis
eFigure 6. Simple vs Radical (Extended) Hysterectomy in Patients With IA2 and IB1 Cervical Cancer With a Tumor Smaller Than 2 cm
eTable 4. The Raw Data for Graph 7A
eFigure 7. Fertility-Sparing Surgery Versus Hysterectomy in Patients With Cervical Cancer and a Tumor Size Smaller Than 2 cm and Who Were 25 to 35 Years Old
eFigure 8. Fertility-Sparing Versus Non-Fertility-Sparing Treatment in Patients With Clinical Stage IA or IC Ovarian Cancer Aged Less 40

This supplemental material has been provided by the authors to give readers additional information about their work.

© 2025 Kanbergs A et al. *JAMA Network Open*.

eTable 1 Cohorts included in our analyses and the rationale for their selection

De-escalation Variable	Cancer Type	Cohort Included in Analysis	Current NCCN Guidelines	Rationale
**Surgical Approach**	Endometrial Cancer	Patients with the indicated cancers who underwent surgical treatment for any stage disease	Th or BSO and LN assessment may be performed by any surgical route; however, MIS is the standard treatment for patients with uterine-confined disease.	Patients with any stage of the indicated cancers were considered because surgery has remained the primary treatment recommendation, regardless of the surgical approach (Figure 2).
	Ovarian Cancer		An open laparotomy should be used for most patients with suspected malignant ovarian cancer who have a planned surgical staging procedure, a primary debulking procedure, an interval debulking procedure, or secondary cytoreduction.<br><br>For select patients, MIS may be employed by an experienced surgeon to manage early-stage disease or for interval debulking procedures. Patients who are unable to undergo MIS for optimal debulking should undergo an open procedure instead.	While we considered patients with any stage of the indicated cancers when observing the overall trends (Figure 2), we conducted a subgroup analysis due to evidence of the increasing use of MIS for patients with early-stage disease and for interval debulking surgeries for patients with advance-stage disease (Appendix 4). This focused analysis enabled a better understanding for how the early or advanced stage impacts these evolving trends.
	Cervical Cancer		An open abdominal approach is the standard approach for radical hysterectomy.	The indication for open surgery changed in 2018 following the LACC publication.[1] To capture the overall trends, both laparoscopic and open approaches were included. A subgroup analysis was also conducted, which focused on patients with early-stage disease where surgical treatment is primarily indicated (Appendix 3).
**LN Assessment**	Endometrial Cancer	Patients who underwent an LND (SLND, LND, or SLND +LND) and had clinical stage I or II disease	LN assessment is indicated for apparent uterine-confined endometrial stage I and II carcinoma, but SLN mapping is preferred.	The cohort analyzed is consistent with NCCN guidelines. Additionally, we analyzed SLN mapping based on histological risk groups, particularly considering that evidence initially supported its use for low-risk patients, with evidence supporting use for other risk groups emerging in later years (Figure 3B).[2]
		Patients who underwent pelvic or pelvic and para-aortic LN dissection and had clinical stage 1 or 2 disease	Para-aortic nodal evaluation may also be used for cancer staging in patients with high-risk tumors, such as deeply invasive lesions; tumors with high-grade histology; and serous carcinoma, clear cell carcinoma, or carcinosarcoma tumors.	The cohort analyzed is consistent with NCCN guidelines. Additionally, we aimed to analyze the trends over the years based on histological risk groups (Appendix 6).
	Cervical Cancer	Patients who underwent an LND (SLND, LND, or	Sentinel or pelvic lymphadenectomy LN assessment is indicated for patients who undergo	The cohort analyzed is consistent with NCCN guidelines (Figure 3A).

© 2025 Kanbergs A et al. *JAMA Network Open*.

		SLND + LND) and had clinical stage I or IIA1 disease	hysterectomy with clinical stage IA1 with LVSI, IA2, IB1, IB2, or IIA1 disease.	
		Patients with cervical cancer (any stage) who underwent pelvic or pelvic with para-aortic LND	Pelvic node sampling is recommended for patients with IA1 and IA2, even with LVSI, disease, and para-aortic LN evaluation is not indicated.<br><br>Para-aortic LN evaluation can be considered for patients with IB1, IB2, IIA1 disease.<br><br>It is category 2B to perform para-aortic LN evaluation for patients with stage IB3 and IIA2, IIB, III, or IVA disease.	The FIGO staging system for cervical cancer was updated in 2018. Prior to this update, LN involvement was not part of the staging criteria. Therefore, the analysis by stages could not be performed. Instead, the overall trends among all stages were analyzed (Appendix 5).
	Vulvar Cancer	Patients who underwent a LN assessment and had clinical stage IB or II disease with a tumor smaller than 4 cm	LN assessment for patients with 1A disease is not indicated, given risk of LN metastasis is <1%.<br><br>Candidates for SLND include patients with a negative clinical groin examination and/or negative imaging, and a primary unifocal vulvar tumor smaller than 4 cm.	The cohort analyzed is consistent with NCCN guidelines (Figure 3C).
**Organs or Structures preserved**	Endometrial Cancer	Patients with clinical stage IA endometrial cancer, endometrioid histology (excluding grade 3), and who were younger than 40 years old, who underwent either hysterectomy without oophorectomy or hysterectomy with oophorectomy	Ovarian preservation may be safe in select premenopausal patients who are premenopausal with early-stage endometrioid cancer, normal-appearing ovaries, and no family history of breast/ovarian cancer or Lynch syndrome. Salpingectomy is recommended for all patients.	We selected the age of 40 to capture the group of patients most likely to benefit from retention of ovaries from either a fertility-sparing or hormonal benefit standpoint. We also excluded patients with grade 3 tumors because these patients are not the best candidates for preserving ovaries due to the high-risk features of such tumors (Figure 5).

© 2025 Kanbergs A et al. *JAMA Network Open*.

	Cervical Cancer	Patients with clinical stage IA2 or IB1 disease and tumor smaller than 2 cm with invasive histology, who underwent a LN assessment, and who underwent any type of hysterectomy	Modified RH is indicated for patients with stage IA1 and IA2 with LVSI.<br><br>RH is indicated for patients with for IB1 who do not meet conservative surgery criteria and Stage IB2 Stage IIA1.	We selected our study population based on inclusion criteria from prospective studies evaluating oncologic outcomes that compare simple and radical hysterectomy. Notably, the SHAPE trial, a recent randomized controlled study, compared modified radical hysterectomy with simple hysterectomy for patients with low-risk early-stage cervical cancer 2 cm or smaller (Appendix 7).[3]
**Fertility-Sparing Surgery**	Ovarian Cancer	Patients with clinical stage IA or IC ovarian cancer, who were age younger than 40 years old and who underwent standard or FSS	Fertility-sparing surgery may be performed (if technically feasible) if the intraoperative frozen section results are positive for apparent early-stage tumors and/or low-risk tumors (ie, malignant germ cell tumors, borderline epithelial tumors, clinical stage I epithelial ovarian tumors, clinical stage I mucinous tumors, or clinical stage I sex cord-stromal tumors).	We excluded patients with clinical IB disease, as this treatment of this disease indicates the removal of bilateral ovaries, which is not consistent with our definition of fertility-sparing treatment (Appendix 9).
	Cervical Cancer	Patients who underwent fertility-sparing treatment or hysterectomy, who were younger than 40 years old, and who had a tumor smaller than 2 cm	Fertility-sparing surgery can be considered for patients with clinical IA1-IB1 disease and patients with select 1B2 disease.<br><br>Fertility-sparing surgery for stage IB has been most validated for tumors 2 cm or smaller.	The cohort analyzed is consistent with NCCN guidelines (Figure 6)<br><br>Additionally, we analyzed trends across the age interval 25 to 35, given this age group has the greatest fertility potential (Appendix 7).

For this paper's purpose, surgical de-escalation was defined as using less invasive surgical approaches or reducing the extent of surgery (including the removal of fewer organs, anatomical structures, or LNs). When selecting our cohorts for the trend analyses, we generally chose those that were consistent with category 2B (ie, NCCN states that the intervention is appropriate based on lower-level evidence) or greater NCCN guidelines. However, when we have deviated from this practice, we have provided a rationale as to why.

All histologies were included in analysis unless otherwise noted

Abbreviations
BSO, bilateral salpingo-oophorectomy; FIGO, International Federation of Gynecology and Obstetrics; FSS, Fertility-Sparing Surgery; LN, lymph node; LND, lymph node dissection; LVSI, lymph-vascular space invasion; MIS, minimally invasive surgery; NCCN, National Comprehensive Cancer Network; RCT, randomized clinical trial; RH, radical hysterectomy; SLND, sentinel lymph node dissection; TH, total hysterectomy

© 2025 Kanbergs A et al. *JAMA Network Open*.

eTable 2 NCBD variables/codes used in study analyses

a) **Codes used to define surgical approach and sentinel, complete, and para-aortic lymphadenectomy**

Cancer Type	Primary Site Codes	Surgical Approach Codes	Sentinel Versus Complete Lymphadenectomy Codes	Pelvic Versus Pelvic with Para-aortic Lymphadenectomy Codes
Endometrial Cancer	C54.0-C55.9	RX_HOSP_SURG_APPR_2010<br>Minimally invasive codes: 1, 3<br>Open codes: 5<br>Conversion codes: 2, 4	RX_SUMM_SCOPE_REG_LN_2012<br>1. SLN: code 2<br>2. Lymphadenectomy only: code 5<br>3. Both SLN+LND: code 6 and 7<br><br>REGIONAL_NODES_EXAMINED<br>1. SLN: codes 1-3<br>2. Lymphadenectomy: codes 4-90	For patients who underwent a complete lymphadenectomy (RX_SUMM_SCOPE_REG_LN_2012 codes 5-7), we then evaluated pelvic versus pelvic + para-aortic lymph node dissection using the CS-SSF available at https://staging.seer.cancer.gov/cs/list/02.05.50/ .<br><br>CS-SSF 4: number of examined pelvic nodes<br>CS-SSF 6: number of examined para-aortic nodes<br>1. Pelvic: CS Site-Specific Factor 4 = 005-089, 090, 097 + CS Site-Specific Factor 6 = 000<br>2. Pelvic + para-aortic: CS Site-Specific Factor 4 = 005-089, 090, 097 + CS Site-Specific Factor 6 = 001-089, 090, 097
Ovarian Cancer (Including Fallopian Tubes Cancer)	C56.9 and C57.0	RX_HOSP_SURG_APPR_2010<br>Minimally invasive codes: 1, 3<br>Open codes: 5<br>Conversion codes: 2, 4	Not applicable	Not applicable
Cervical Cancer	C53.0-C53.9	RX_HOSP_SURG_APPR_2010<br>Minimally invasive codes: 1, 3<br>Open codes: 5<br>Conversion codes: 2, 4	RX_SUMM_SCOPE_REG_LN_2012<br>1. SLN: code 2<br>2. Lymphadenectomy only: code 5<br>3. Both SLN + LND: code 6 and 7<br><br>REGIONAL_NODES_EXAMINED<br>1. SLN: codes 1-3<br>2. Lymphadenectomy: codes 4-90	For patients who underwent a complete lymphadenectomy (RX_SUMM_SCOPE_REG_LN_2012 codes 5-7), we then evaluated pelvic versus pelvic + para-aortic lymph node dissection using the CS-SSF available at https://staging.seer.cancer.gov/cs/list/02.05.50/<br><br>Regional nodes examined:

© 2025 Kanbergs A et al. *JAMA Network Open*.

				CS-SSF 3: Assessment Method of Pelvic Nodal Status<br>CS-SSF 4: Para-Aortic Nodal Status<br>CS-SSF 5: Assessment Method of Para-Aortic Nodal Status<br><br>Pelvic:<br>1. CS-SSF 3 = 040 + CS site-specific factor 5 = 000, 998 OR<br>2. Regional nodes examined = 005-089, 090, 097 + CS site-specific factor 5 = 000, 998<br>Pelvic + para-aortic:<br>1. CS-SSF 4 = 005-089, 090, 097 + CS site-specific factor 5 = 040 OR<br>2. Regional nodes examined = 005-089, 090, 097 + CS site-specific factor 5 = 040
Vulvar Cancer	C51.0-C51.2, C51.8-C51.9	Not applicable	RX_SUMM_SCOPE_REG_LN_2012<br><br>1. SLN: code 2<br>2. Lymphadenectomy only: code 5<br>3. Both SLN+LND: code 6 and 7<br><br>REGIONAL_NODES_EXAMINED<br>1. SLN: codes 1-3<br>2. Lymphadenectomy: codes 4-90	Not applicable

CS-SSF Collaborative State Site-Specific Factors; SLN, sentinel lymph node; LND, lymph node dissection

© 2025 Kanbergs A et al. *JAMA Network Open*.

b) Specific cancer surgical procedures and codes

Cancer Type	Surgical Procedure	Codes
Endometrial Cancer	Ovarian preservation<br>Hysterectomy With removal of ovaries	Codes 40 and 66<br>Codes 50 and 67
Ovarian Cancer	Standard surgery<br>Debulking surgery with intestinal procedures<br>Debulking surgery with urinary procedures with or without intestinal procedures	Codes 52 and 57<br>Codes 61 and 72<br>Codes 62, 63, and 71
	Neoadjuvant chemotherapy<br>Adjuvant chemotherapy	Codes 2 and 4<br>Code 3
	Fertility-sparing surgery (uterus and one ovary preservation)<br>Non–fertility-sparing surgery	Codes 27 and 36,<br>Codes 28, 50, 51, 52, and 57
Cervical Cancer	Simple hysterectomy<br>Radical hysterectomy	Codes 30 and 40<br>Codes 51, 52, 53, and 54
	Fertility-sparing procedures	Codes 15, 27, 24, 29, and 28
Vulvar Cancer	Radical vulvar surgery<br>Simple vulvar surgery	Codes 40, 50, and 60<br>Codes 20 and 30

© 2025 Kanbergs A et al. *JAMA Network Open*.

eTable 3 Patient Characteristics

	All patients (n=1,218,490)	Cervical (n=166,779)	Endometrial (n=686,458)	Ovarian (n=301,123)	Vulvar (n=64,130)
**Diagnosis age (year, SD)**	61.2 ± 13.7	50.9 ± 14.8	62.8 ± 11.8	62.4 ± 14.3	66.1 ± 15.0
**Cancer stage**					
I	624922 (51.3%)	70670 (42.4%)	457434 (66.6%)	65527 (21.8%)	31291 (48.8%)
II	93898 (7.7%)	25928 (15.5%)	34564 (5.0%)	25069 (8.3%)	8337 (13.0%)
III	215628 (17.7%)	29910 (17.9%)	68579 (10.0%)	108441 (36.0%)	8698 (13.6%)
IV	148534 (12.2%)	22337 (13.4%)	51124 (7.4%)	70083 (23.3%)	4990 (7.8%)
Unknown	135508 (11.1%)	17934 (10.8%)	74757 (10.9%)	32003 (10.6%)	10814 (16.9%)
**Grade**					
1	310788 (25.5%)	17502 (10.5%)	255894 (37.3%)	21823 (7.2%)	15569 (24.3%)
2	252068 (20.7%)	52658 (31.6%)	147757 (21.5%)	30159 (10.0%)	21494 (33.5%)
3	279026 (22.9%)	47090 (28.2%)	127682 (18.6%)	96487 (32.0%)	7767 (12.1%)
Unknown	376608 (30.9%)	49529 (29.7%)	155125 (22.6%)	152654 (50.7%)	19300 (30.1%)
**Race ethnicity**					
NH White	934735 (76.7%)	107603 (64.5%)	532536 (77.6%)	239906 (79.7%)	54690 (85.3%)
Black	130137 (10.7%)	25400 (15.2%)	74604 (10.9%)	25130 (8.3%)	5003 (7.8%)
Asian/American Ind	45936 (3.8%)	8190 (4.9%)	24711 (3.6%)	12078 (4.0%)	957 (1.5%)
Hispanic	87816 (7.2%)	22431 (13.4%)	43301 (6.3%)	19488 (6.5%)	2596 (4.0%)
Unknown	19866 (1.6%)	3155 (1.9%)	11306 (1.6%)	4521 (1.5%)	884 (1.4%)
Insurance					
**Private**	552499 (45.3%)	78753 (47.2%)	319929 (46.6%)	133302 (44.3%)	20515 (32.0%)
Medicaid and other	114599 (9.4%)	37776 (22.7%)	48277 (7.0%)	22794 (7.6%)	5752 (9.0%)
Medicare	480646 (39.5%)	32375 (19.4%)	285387 (41.6%)	128346 (42.6%)	34538 (53.9%)
None	49565 (4.1%)	13740 (8.2%)	22323 (3.3%)	11199 (3.7%)	2303 (3.6%)
Unknown	21181 (1.7%)	4135 (2.5%)	10542 (1.5%)	5482 (1.8%)	1022 (1.6%)
**Charlson score**					
Score 0	931955 (76.5%)	141404 (84.8%)	507284 (73.9%)	237454 (78.9%)	45813 (71.4%)
Score 1	210403 (17.3%)	18785 (11.3%)	133121 (19.4%)	46233 (15.4%)	12264 (19.1%)

© 2025 Kanbergs A et al. *JAMA Network Open*.

Score 2	50501 (4.1%)	4179 (2.5%)	31057 (4.5%)	11525 (3.8%)	3740 (5.8%)
Score 3+	25631 (2.1%)	2411 (1.4%)	14996 (2.2%)	5911 (2.0%)	2313 (3.6%)
**Facility type**					
Community Cancer Program	50039 (4.1%)	7811 (4.7%)	27075 (3.9%)	12950 (4.3%)	2203 (3.4%)
Comprehensive community	427862 (35.1%)	54007 (32.4%)	243658 (35.5%)	108718 (36.1%)	21479 (33.5%)
Academic/research	488190 (40.1%)	73426 (44.0%)	269869 (39.3%)	117556 (39.0%)	27339 (42.6%)
Integrated Network	252397 (20.7%)	31535 (18.9%)	145855 (21.2%)	61898 (20.6%)	13109 (20.4%)
Unknown	2 (0)	0 (0.0%)	1 (0.0%)	1 (0.0%)	0 (0.0%)
**Urban/rural**					
Metropolitan	995252 (81.7%)	136387 (81.8%)	561249 (81.8%)	247344 (82.1%)	50272 (78.4%)
Urban	159215 (13.1%)	22305 (13.4%)	89764 (13.1%)	37113 (12.3%)	10033 (15.6%)
Rural	19631 (1.6%)	2593 (1.6%)	11101 (1.6%)	4717 (1.6%)	1220 (1.9%)
Unknown	44392 (3.6%)	5494 (3.3%)	24344 (3.5%)	11949 (4.0%)	2605 (4.1%)

© 2025 Kanbergs A et al. *JAMA Network Open*.

eFigure 1 Patients who underwent surgical treatment of gynecologic cancer stratified by early (stage I and II) and late (stage III and IV) stage

A. Early stage (I and II)

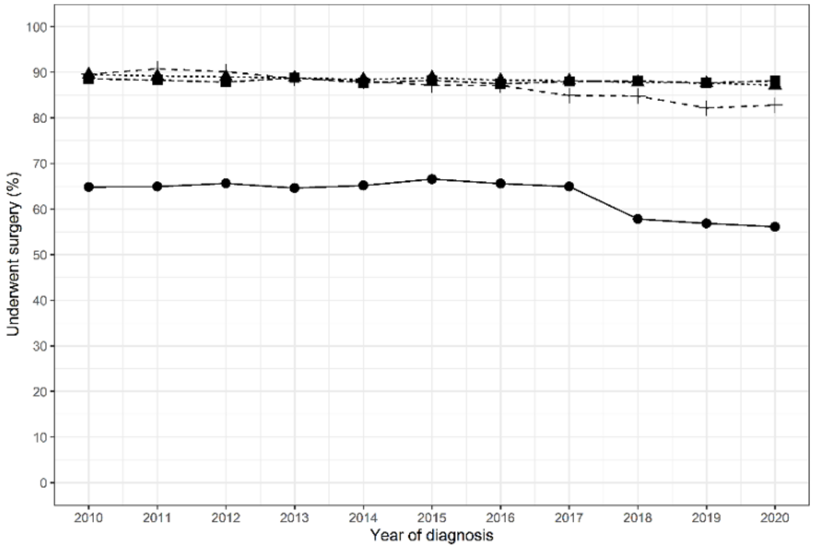

Year	Cervical (%)	Endometrial (%)	Ovarian (%)	Vulvar2 (%)
2010	64.9	89.5	88.6	89.5
2011	64.9	89.2	88.3	90.8
2012	65.6	89.0	87.8	90.1
2013	64.6	88.8	88.8	88.7
2014	65.2	88.4	87.7	88.1
2015	66.5	88.8	88.2	87.1
2016	65.6	88.3	87.5	87.1
2017	64.9	88.2	88.0	84.9
2018	57.8	87.8	88.1	84.8
2019	56.9	87.6	87.7	82.2
2020	56.1	87.1	88.1	82.8
Average annual percentage change	-1.4%	-0.2%	-0.1%	-1.0%
95% CI.	-1.7% to -1.1%	-0.4% to -0.1%	-0.3% to 0.2%	-1.4% to -0.6%

B. late stage (III and IV)

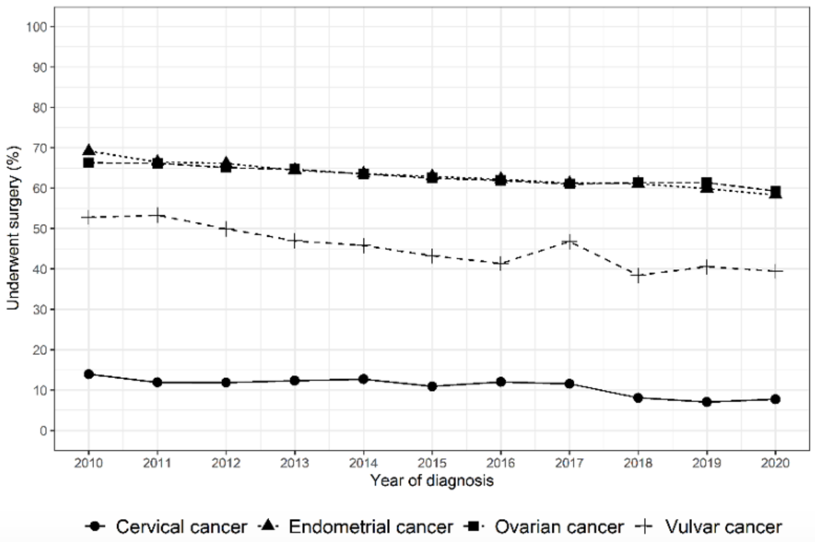

Year	Cervical (%)	Endometrial (%)	Ovarian (%)	Vulvar2 (%)
2010	13.9	69.2	66.3	52.8
2011	11.9	66.5	66.1	53.3
2012	11.8	66.1	65.1	49.9
2013	12.3	64.5	64.7	47.0
2014	12.7	63.6	63.5	45.8
2015	10.9	62.9	62.5	43.2
2016	12.0	62.2	61.9	41.4
2017	11.6	61.2	61.0	46.7
2018	8.1	61.1	61.4	38.4
2019	7.0	59.9	61.4	40.5
2020	7.7	58.3	59.2	39.4
Average annual percentage change	-5.1%	-1.5%	-1.1%	-3.1%
95% CI.	-6.0% to -4.1%	-1.7% to -1.2%	-1.3% to -0.8%	-4.0% to -2.1%

© 2025 Kanbergs A et al. *JAMA Network Open*.

eFigure 2 Minimally invasive surgery (MIS), open surgery, or conversion-to-open surgery (conversion) among patients with cervical, endometrial, or ovarian cancer and stage I or II disease

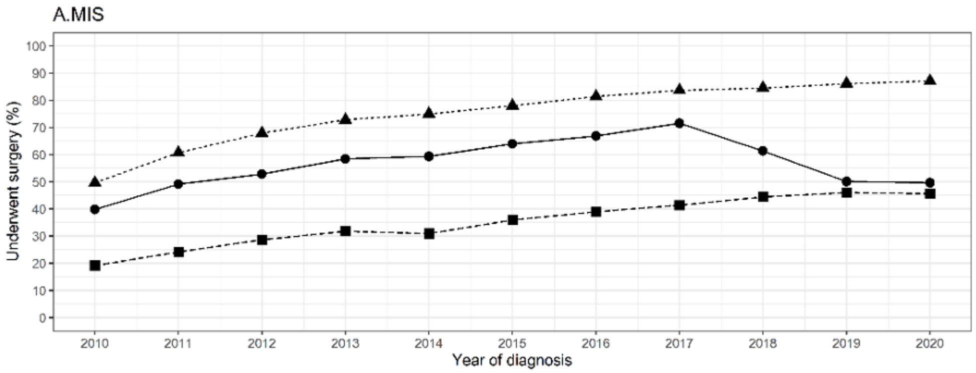

Year	Cervical (%)	Endometrial (%)	Ovarian (%)
2010	39.7	49.6	19.1
2011	49.1	60.7	24.2
2012	52.8	67.9	28.6
2013	58.4	72.8	31.7
2014	59.3	75.0	30.9
2015	64.0	78.0	35.9
2016	66.8	81.4	38.9
2017	71.5	83.7	41.3
2018	61.3	84.5	44.4
2019	50.1	86.1	46.0
2020	49.6	87.1	45.6
Average annual percentage change	2.0%	4.5%	8.1%
95% CI.	1.6% to 2.4%	4.3% to 4.6%	7.6% to 8.6%

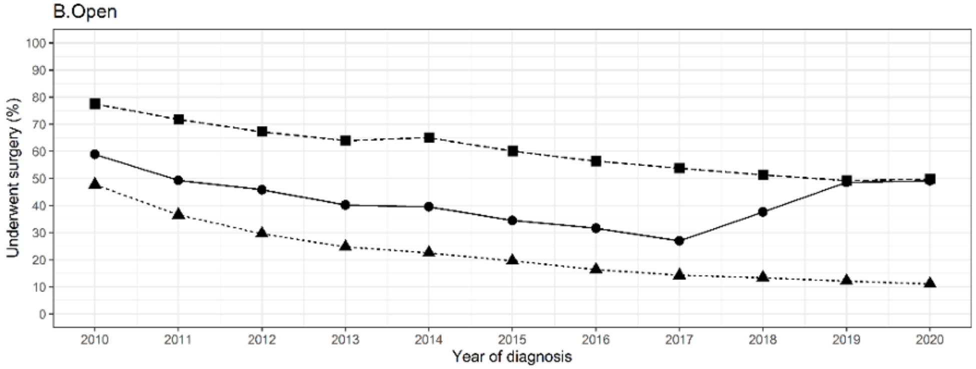

Year	Cervical (%)	Endometrial (%)	Ovarian (%)
2010	58.8	47.7	77.5
2011	49.3	36.5	71.7
2012	45.8	29.6	67.1
2013	40.2	24.7	63.9
2014	39.5	22.5	65.0
2015	34.5	19.5	60.0
2016	31.6	16.3	56.3
2017	26.9	14.2	53.7
2018	37.6	13.4	51.3
2019	48.5	12.1	49.2
2020	49.1	11.1	49.8
Average annual percentage change	-2.6%	-13.9%	-4.5%
95% CI.	-3.1% to -2.2%	-14.1% to -13.7%	-4.9% to -4.2%

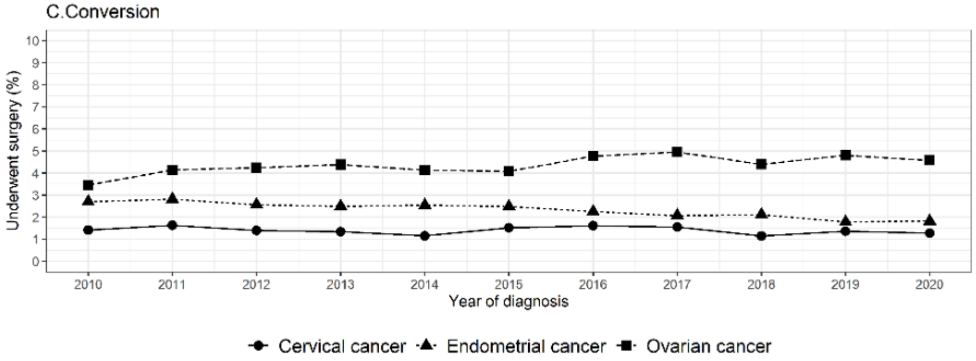

Year	Cervical (%)	Endometrial (%)	Ovarian (%)
2010	1.4	2.7	3.5
2011	1.6	2.8	4.1
2012	1.4	2.6	4.2
2013	1.3	2.5	4.4
2014	1.1	2.5	4.1
2015	1.5	2.5	4.1
2016	1.6	2.3	4.8
2017	1.5	2.1	5.0
2018	1.1	2.1	4.4
2019	1.4	1.8	4.8
2020	1.3	1.8	4.6
Average annual percentage change	-1.0%	-4.3%	2.2%
95% CI.	-3.6% to 1.6%	-5.0% to -3.6%	0.9% to 3.6%

© 2025 Kanbergs A et al. *JAMA Network Open*.

eFigure 3. Minimally invasive surgery (MIS), open surgery, or conversion-to-open surgery (conversion) among patients with cervical, endometrial, or ovarian cancer and stage III or IV disease

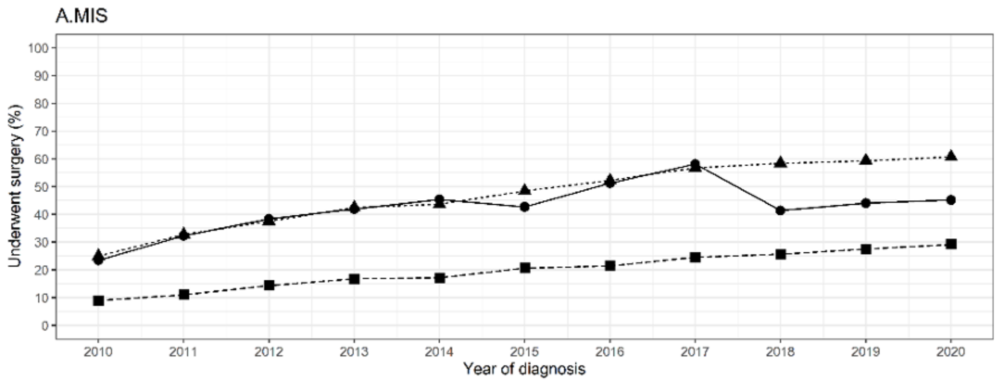

Year	Cervical (%)	Endometrial (%)	Ovarian (%)
2010	23.4	24.9	8.9
2011	32.3	32.7	11.0
2012	38.3	37.5	14.3
2013	41.9	42.4	16.7
2014	45.3	43.6	17.1
2015	42.6	48.4	20.6
2016	51.2	52.2	21.4
2017	58.1	56.7	24.6
2018	41.4	58.4	25.6
2019	44.0	59.3	27.5
2020	45.1	60.7	29.1
Average annual percentage change	5.7%	7.7%	11.0%
95% CI.	4.0% to 7.4%	7.2% to 8.1%	10.4% to 11.6%

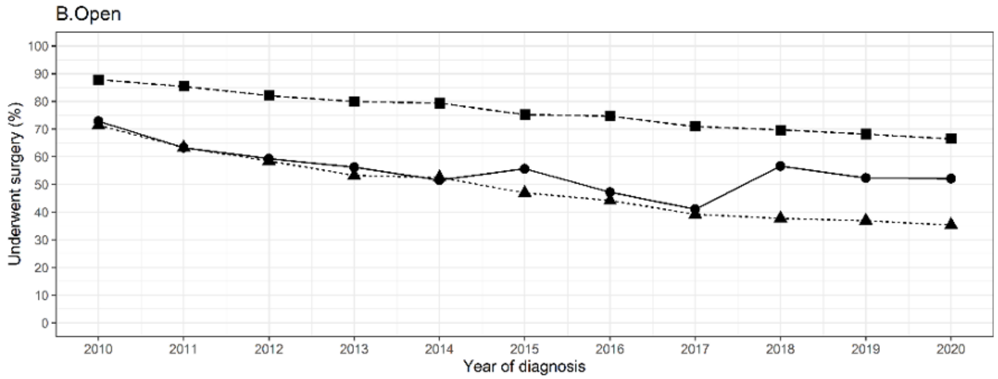

Year	Cervical (%)	Endometrial (%)	Ovarian (%)
2010	72.8	71.3	87.8
2011	63.2	63.3	85.3
2012	59.3	58.5	82.0
2013	56.2	53.2	79.9
2014	51.6	52.5	79.3
2015	55.6	46.9	75.2
2016	47.2	44.2	74.6
2017	41.0	39.2	70.9
2018	56.5	37.7	69.6
2019	52.3	36.8	68.1
2020	52.1	35.3	66.4
Average annual percentage change	-3.9%	-6.9%	-2.8%
95% CI.	-5.3% to -2.5%	-7.3% to -6.6%	-3.0% to -2.5%

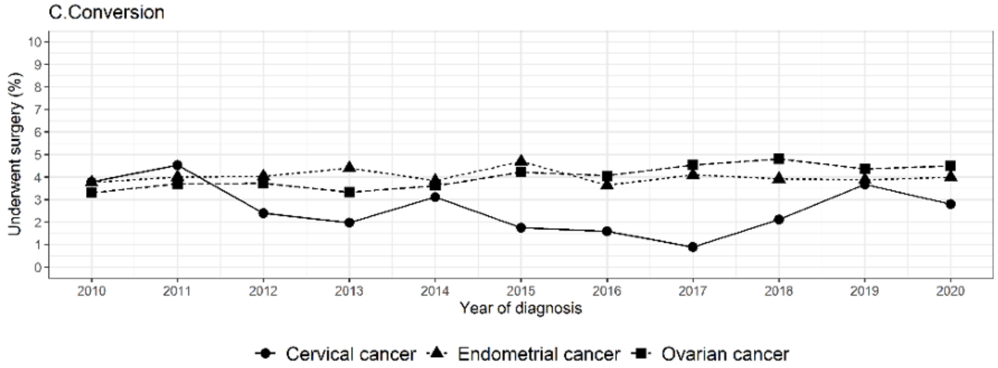

Year	Cervical (%)	Endometrial (%)	Ovarian (%)
2010	3.8	3.8	3.3
2011	4.5	4.0	3.7
2012	2.4	4.0	3.7
2013	2.0	4.4	3.3
2014	3.1	3.8	3.6
2015	1.7	4.7	4.2
2016	1.6	3.6	4.1
2017	0.9	4.1	4.5
2018	2.1	3.9	4.8
2019	3.7	3.9	4.4
2020	2.8	4.0	4.5
Average annual percentage change	-7.1%	-0.2%	3.4%
95% CI.	-13.2% to -0.5%	-1.5% to 1.2%	2.3% to 4.6%

© 2025 Kanbergs A et al. *JAMA Network Open*.

eFigure 4 Pelvic or pelvic + para-aortic lymph node dissection in patients with any cervical cancer

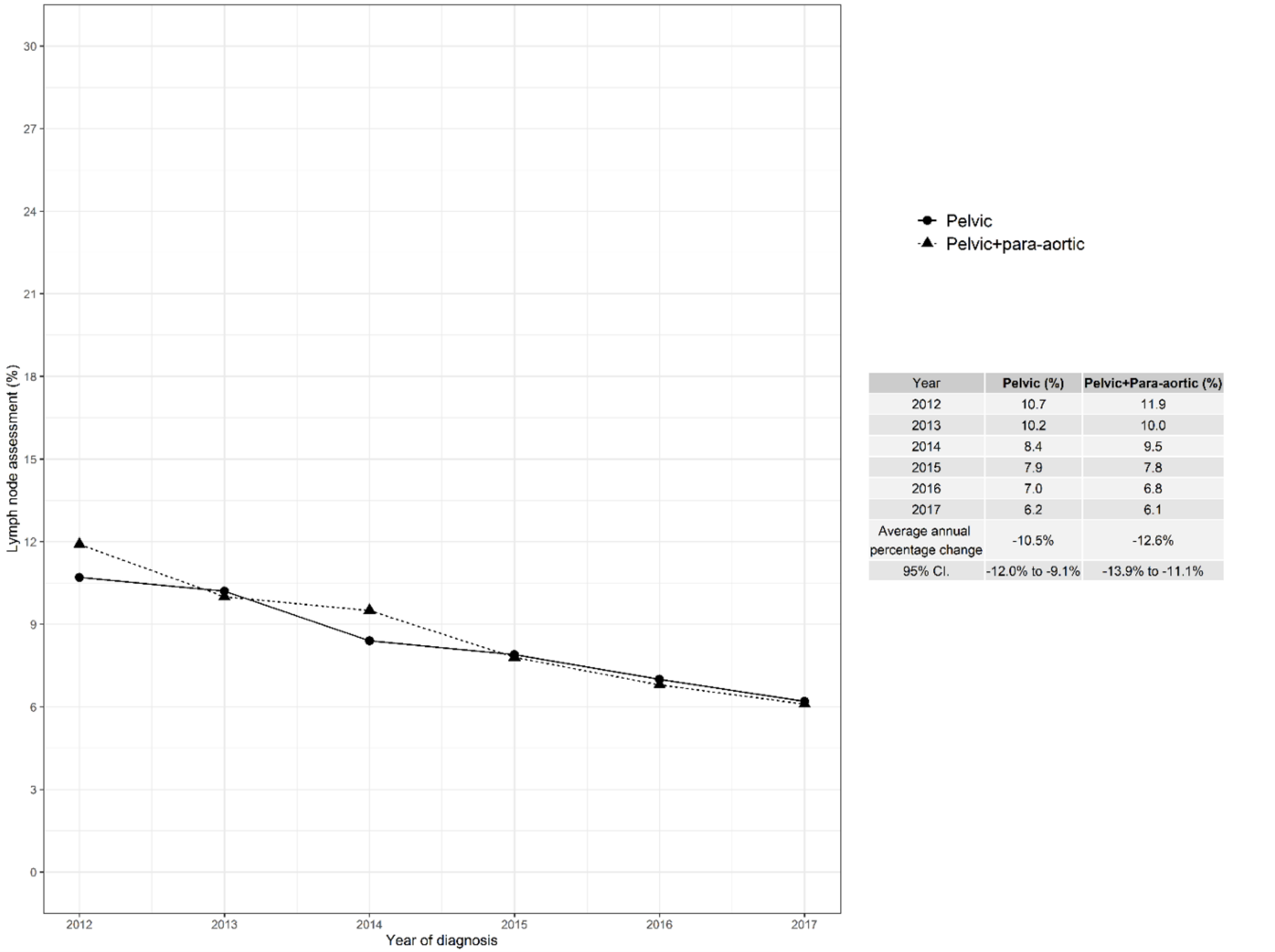

Year	Pelvic (%)	Pelvic+Para-aortic (%)
2012	10.7	11.9
2013	10.2	10.0
2014	8.4	9.5
2015	7.9	7.8
2016	7.0	6.8
2017	6.2	6.1
Average annual percentage change	-10.5%	-12.6%
95% CI.	-12.0% to -9.1%	-13.9% to -11.1%

© 2025 Kanbergs A et al. *JAMA Network Open*.

eFigure 5 Para-aortic lymph node assessment in patients with early-stage endometrial cancer stratified by risk factors for nodal metastasis

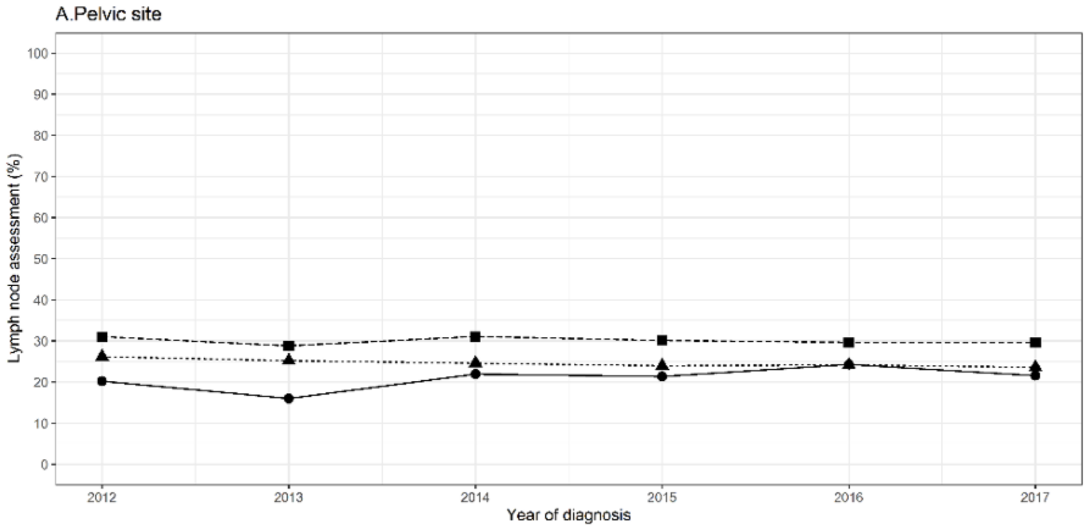

Year	Stage IA, Grade 1 or 2 endometrioid histology (%)	Stage IB, Grade 1 or 2 endometrioid histology (%)	Stage I-II, High-risk histology (%)
2012	26.2	31.0	20.2
2013	25.2	28.8	16.0
2014	24.6	31.1	21.9
2015	24.0	30.2	21.4
2016	24.2	29.6	24.2
2017	23.6	29.6	21.6
Average annual percentage change	-2.0%	-0.4%	4.3%
95% CI.	-3.5% to -0.5%	-3.6% to 2.8%	-3.3% to 12.6%

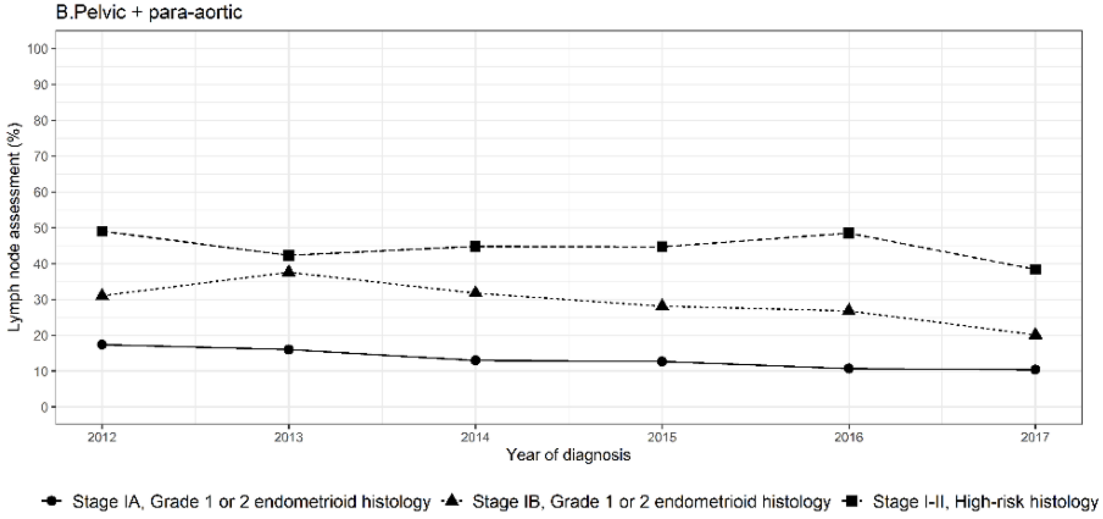

Year	Stage IA, Grade 1 or 2 endometrioid histology (%)	Stage IB, Grade 1 or 2 endometrioid histology (%)	Stage I-II, High-risk histology (%)
2012	17.4	31.0	49.0
2013	16.0	37.6	42.3
2014	13.0	31.7	44.7
2015	12.7	28.1	44.7
2016	10.7	26.8	48.5
2017	10.3	20.1	38.3
Average annual percentage change	-10.6%	-8.1%	-2.2%
95% CI.	-12.5% to -8.7%	-11.0% to -5.0%	-7.2% to 3.0%

© 2025 Kanbergs A et al. *JAMA Network Open*.

eFigure 6. Simple vs radical (Extended) Hysterectomy in patients with IA2 and IB1 cervical cancer with a tumor smaller then 2 cm

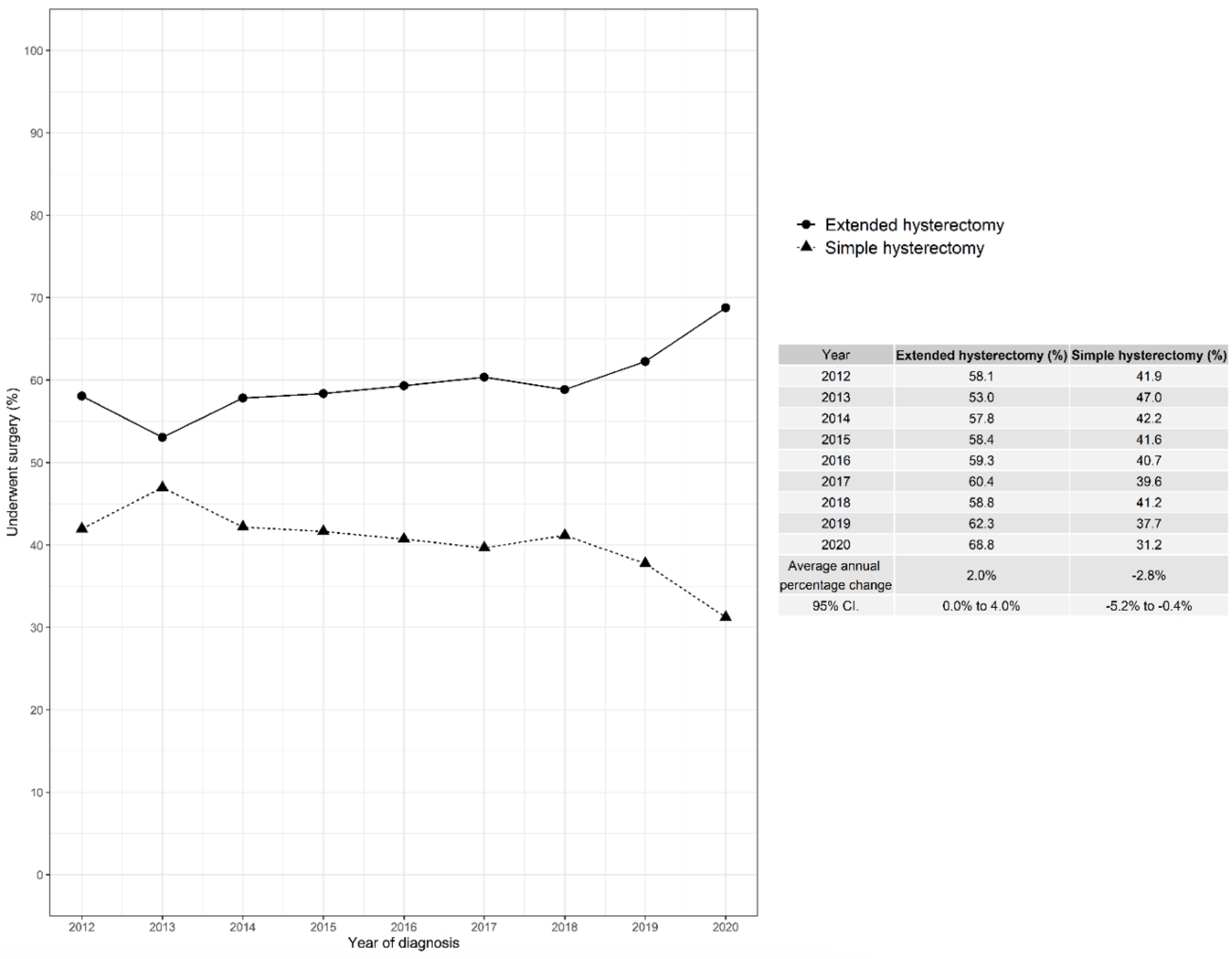

Year	Extended hysterectomy (%)	Simple hysterectomy (%)
2012	58.1	41.9
2013	53.0	47.0
2014	57.8	42.2
2015	58.4	41.6
2016	59.3	40.7
2017	60.4	39.6
2018	58.8	41.2
2019	62.3	37.7
2020	68.8	31.2
Average annual percentage change	2.0%	-2.8%
95% CI.	0.0% to 4.0%	-5.2% to -0.4%

© 2025 Kanbergs A et al. *JAMA Network Open*.

eTable 4. The Raw Data for Graph 7A

Year	No of Patients	Extended Hysterectomy	Simple Hysterectomy
2012	298	173 (58.1)	125 (42.0)
2013	313	166 (53.0)	147 (47.0)
2014	332	192 (57.8)	140 (42.1)
2015	317	185 (58.4)	132 (41.6)
2016	371	220 (59.3)	151 (40.7)
2017	338	204 (60.4)	134 (39.6)
2018	277	163 (58.8)	114 (41.2)
2019	257	160 (62.3)	97 (37.7)
2020	205	141 (68.8)	64 (31.2)

Patients with the following characteristics were included in this analysis: clinical stage 1a2 and 1b1 cervical cancer, invasive histology, tumor smaller than 2 cm, and underwent lymph node assessment and any type of hysterectomy.

© 2025 Kanbergs A et al. *JAMA Network Open*.

eFigure 7 Fertility-sparing surgery versus hysterectomy in patients with cervical cancer and a tumor size smaller than 2 cm and who were 25 to 35 years old.

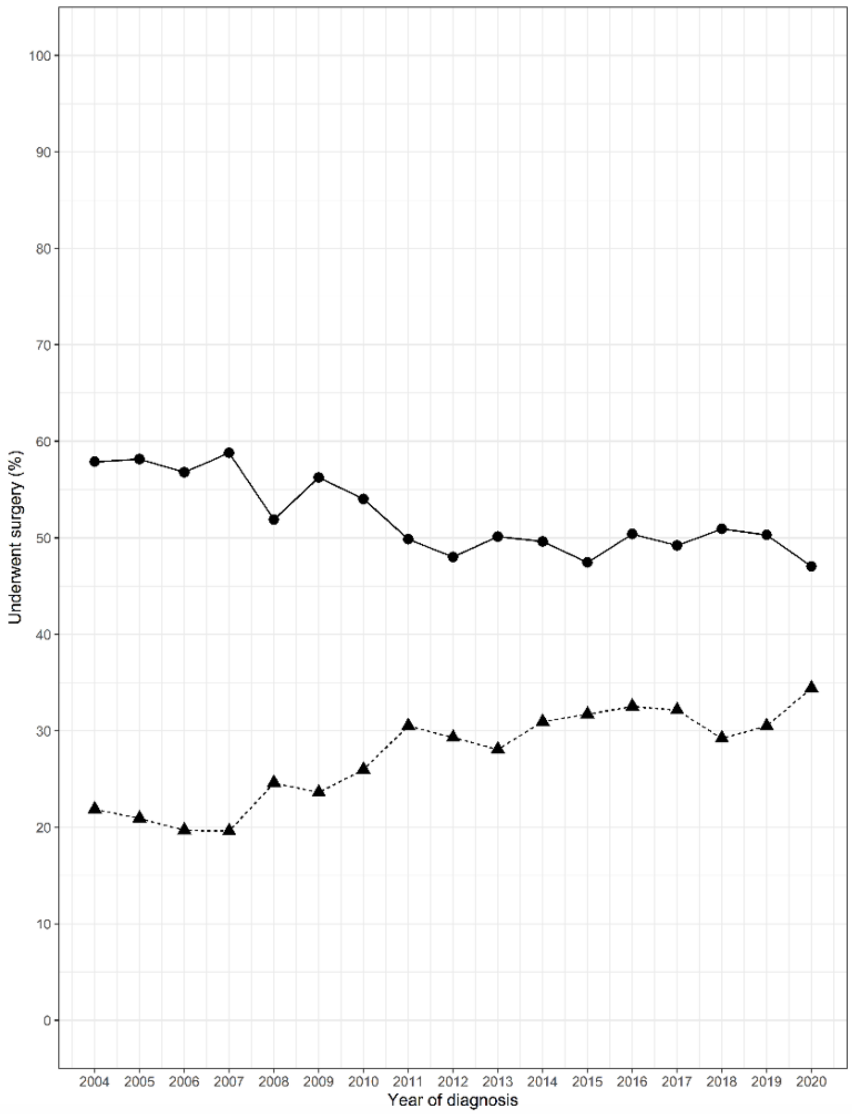

Year	Fertility-sparing surgery (%)	Any type of hysterectomy (%)
2004	21.9	57.9
2005	20.9	58.2
2006	19.7	56.8
2007	19.6	58.8
2008	24.6	51.9
2009	23.6	56.3
2010	26.0	54.0
2011	30.5	49.9
2012	29.3	48.0
2013	28.1	50.1
2014	30.9	49.6
2015	31.7	47.4
2016	32.5	50.4
2017	32.1	49.2
2018	29.2	50.9
2019	30.5	50.3
2020	34.4	47.0
Average annual percentage change	3.2%	-1.2%
95% CI.	2.2% to 4.1%	-1.9% to -0.6%

© 2025 Kanbergs A et al. *JAMA Network Open*.

eFigure 8 Fertility-sparing versus non-fertility-sparing treatment in patients with clinical stage IA or IC ovarian cancer aged less 40

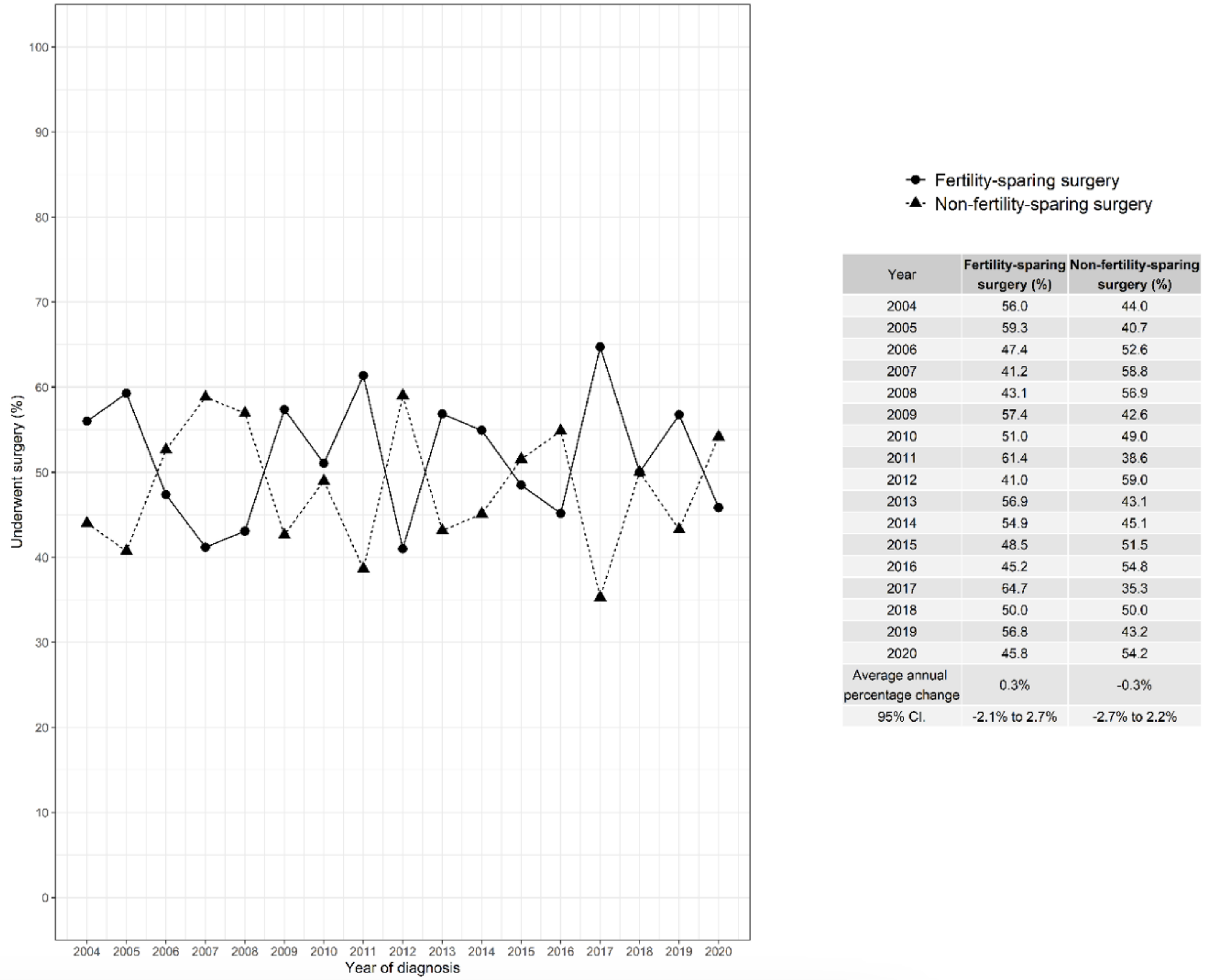

Year	Fertility-sparing surgery (%)	Non-fertility-sparing surgery (%)
2004	56.0	44.0
2005	59.3	40.7
2006	47.4	52.6
2007	41.2	58.8
2008	43.1	56.9
2009	57.4	42.6
2010	51.0	49.0
2011	61.4	38.6
2012	41.0	59.0
2013	56.9	43.1
2014	54.9	45.1
2015	48.5	51.5
2016	45.2	54.8
2017	64.7	35.3
2018	50.0	50.0
2019	56.8	43.2
2020	45.8	54.2
Average annual percentage change	0.3%	-0.3%
95% CI.	-2.1% to 2.7%	-2.7% to 2.2%

© 2025 Kanbergs A et al. *JAMA Network Open*.
