# Supplementary material for: Surgical Deescalation Within Gynecologic Oncology
Source: JAMA Netw Open. 2025 Jan 8;8(1):e2453604. doi: 10.1001/jamanetworkopen.2024.53604 (PMC11811805; doi:10.1001/jamanetworkopen.2024.53604)
Supplement: Supplement 2. — Data Sharing Statement [file jamanetwopen-e2453604-s002.pdf]

Data Sharing Statement

Kanbergs. Surgical Deescalation Within Gynecologic Oncology. *JAMA Netw Open*. Published January 08, 2025. doi:10.1001/jamanetworkopen.2024.53604

Data
Data available: No

Additional Information
Explanation for why data not available: Requests for data will be reviewed individually and approved pending data sharing requirements
